# Threatened sustainability: extractivist tendencies in the forest-based bioeconomy in Finland

**DOI:** 10.1007/s11625-023-01300-9

**Published:** 2023-02-22

**Authors:** Jana R. Holz

**Affiliations:** BMBF Junior Research Group “Mentalities in Flux” (Flumen), Institute of Sociology, Friedrich-Schiller-University, Leutragraben 1, 07743 Jena, Germany

**Keywords:** Forest-based bioeconomy, Forestry extractivism, Biorefinery, Finland, Forest sector, Socio-ecological transformation

## Abstract

Bioeconomy is portrayed by the EU and several national governments as a central element contributing to sustainability strategies and a post-fossil transformation. This paper critically engages with extractivist patterns and tendencies in the forest sector as one of the main bio-based sectors. It argues that despite the official endorsement of circularity and renewability in the forest-based bioeconomy, current developments of modern bioeconomy might threaten sustainability prospects. The Finnish forest-based bioeconomy and one of its well-known showcase projects, the bioproduct mill (BPM) in the municipality of Äänekoski, serve as a case study in this paper. The forest-based bioeconomy in Finland is scrutinized as a potential continuation or consolidation of extractivist patterns, rather than an alternative to these tendencies. The lens of extractivism is applied to identify possible extractivist and unsustainable characteristics of the case study which are discussed along the following dimensions: (A) degree of export orientation and processing, (B) the scale, scope, and speed of extraction, (C) socio-economic and environmental impacts, and (D) subjective relations to nature. The extractivist lens provides analytical value to scrutinizing practices, principles, and dynamics of the contested political field and vision of bioeconomy in the Finnish forest sector. The analysis results in a discussion of latent and manifest social, political, and ecological contradictions within the forest-based bioeconomy in Finland. Based on its analytical lens and the empirical case of the BPM in Äänekoski, it can be concluded that extractivist patterns and tendencies are perpetuated within the Finnish forest-based bioeconomy.

## Introduction

Bioeconomy is being promoted by the EU and several national governments as a sustainable alternative to a fossil-based economy (European Commission 2018a, 2020; Finnish Government 2022). Instead of the extraction of gas, coal, oil, and minerals providing the materials and energy for the economy—and causing unavoidable environmental destruction—a bio-based economy is organized around the principles of circularity and renewability (European Commission 2018a, 2020; Finnish Government 2022). The European Green Deal frames bioeconomy as “a catalyst for systemic change” by tackling “economic, social and environmental aspects” (European Commission 2020). With a win–win scenario of decoupling economic growth from an increase in resource use and emissions by promoting substitution, waste stream management, and biotechnology innovations, bioeconomy policies fit well into current Green Growth narratives (D’Amato et al. 2017). Accordingly, the account of bioeconomy currently dominating bioeconomic politics in the EU is a large-scale, growth-oriented, and highly technologized one (Hausknost et al. 2017). Recent critical accounts on bioeconomy and its role in socio-ecological transformation processes highlight so far missing aspects regarding global and environmental justice (Ramcilovic-Suominen 2022) and fair participation (Holmgren et al. 2021), as well as untenable growth promises (Eversberg et al. 2022a).

Studies applying the heuristic of extractivism to industrial agriculture and bio-based energy production or forestry (so far, mainly to cases in the Global South) support this critical debate. They claim that current dominant bioeconomy programs form a continuation of (fossil) extractivist patterns instead of positively contributing to a socio-ecological transformation toward a sustainable society (Backhouse et al. 2021; Boyer 2019; Ehrnström-Fuentes and Kröger 2018; Landherr et al. 2019; McKay 2017; Tittor 2021; Willow 2019). Following Boyer (2019), current dominant visions of bioeconomy should be framed as a *de-facto alternative form* of extractivism, rather than an alternative to (fossil) extractivism. In this interpretation, bioeconomy implies both a large-scale, high-speed industrial extraction and a vision of ‘nature’ as a resource at the disposal of humans for economic use (Eversberg et al. 2022b; Lühmann 2020). Hence, the negative social, cultural, and environmental effects of fossil extraction and inclusion into global market mechanisms as well as rural development dilemmas might simply continue within a (more) bio-based economy and counteract the sustainability aspirations of bioeconomy actors (D’Amato et al. 2017).

This paper builds on this recent critical research and applies the heuristic of extractivism to the forest-based bioeconomy in Finland by asking whether extractivist patterns and tendencies (McKay 2017; Tittor 2021) are undermining the positive effects of bioeconomy. It focuses on the Finnish forest sector as a case that fits into the dominant vision of bioeconomy, and that is framed by its advocates as a positive contribution to modernizing and ‘greening’ forest utilization (Metsä Group Press Release 2017; Ministry of Agriculture and Forestry Finland 2019; Ministry of Economic Affairs and Employment Finland 2017). ‘Next generation’, high-tech biorefineries are one of the main innovations that the European forest sector relies on to realize the win–win promises of the bioeconomy (European Commission 2018a). The paper studies a showcase project for this approach: the Metsä Fibre/Metsä Group bioproduct mill (BPM) in the municipality of Äänekoski in Central Finland. This BPM won the ‘Mill of the Future’ Award 2020 in the Pulp and Paper Industry (Metsä Fibre 2022a). The Finnish bioeconomy as a whole, as well as local developments in Äänekoski, however, have faced criticism regarding social and environmental aspects (Albrecht 2019; Albrecht and Kortelainen 2020; BIOS 2017a; Eyvindson et al. 2018; Peltomaa 2018). Counter to the sustainability framing brought forward by politics and industry, current developments in the forest sector (also, but not only, in Finland) are characterized as “productivist forest policy” (Kröger and Raitio 2017), plantation forestry (“plantationcentrism”/“plantaasiosentrismiin” (Hyvärinen 2020), as following an “expansion frame” (Toivanen 2021) or plainly as an “expansion of forestry extractivism” (Hanacek et al. 2022).

This paper has two main objectives: It provides an exemplary critical discussion of the contribution of bioeconomy to a sustainable future economy, and an assessment of the potential and limitations of the concept of extractivism for further critical analyses of bioeconomy transitions in the EU. The analysis builds on the existing research into the forest-based bioeconomy in Finland, and is also based on scientific reports, national and EU strategies, as well as exemplary reference to the author’s own qualitative data.

In the section “Dominant understandings of bioeconomy in the EU and Finland” the (forest-based) bioeconomy in Finland and the relevant framing within EU politics are introduced, focusing on contested conceptualizations and the case of the BPM in Äänekoski. Section “Extractivist tendencies and patterns as a heuristic” explains the conceptual heuristic of the paper and discusses the potential of the concept of extractivism for the critical study of forest-based bioeconomy based on four key dimensions of extractivist tendencies and patterns: (A) export orientation and low degree of processing; (B) increasing scale, scope, and speed of extraction; (C) (contradictory) socio-economic and environmental impacts; and (D) extractive relations to nature. Section “Analysis of extractivist tendencies in the Finnish forest-based bioeconomy” analyses the case at hand along these four dimensions. The section “Discussion” condenses the analyses, uncovering social, political, and ecological contradictions in the case. It also suggests applying the extractivist heuristic to further critical analysis of bioeconomic transitions in the EU. The section “Conclusion—unsustainable tendencies in forest-based bioeconomy” summarizes the main discussion points and comes to the result that extractive tendencies and patterns within the forest-based bioeconomy in Finland threaten its possible positive contribution to a sustainable socio-ecological transformation.

## Dominant understandings of bioeconomy in the EU and Finland

Bioeconomy can be defined as an “economy where the basic building blocks for materials, chemicals, and energy are derived from renewable biological resources” (McCormick and Kautto 2013, p. 2589). According to the European Commission (2018a), the primary sectors of agriculture and forestry as well as further sectors related to bio-based services, knowledge, or materials are defined as being part of the bioeconomy. Within the contested field of competing understandings of what a bioeconomy is or should be (Eversberg et al. 2022a; see also: Priefer et al. 2017), different visions are not given equal political or economic relevance (Meyer 2017; Staffas et al. 2013). Disputes about hegemonic interpretations of and policy path for the bioeconomy take place in an arena shaped by alliances among dominant groups and institutions as well as the power of corresponding narrative settings (Birch 2017; Korhonen et al. 2018; Petersen and Krisjansen 2015).

Bioeconomy strategies in the EU present a win–win scenario of decoupling economic growth from an increase in resource use and emissions (D’Amato et al. 2017). The advocates of bioeconomy claim that it has the potential to create “new jobs”, to support the “modernization and strengthening of the EU industrial base” and “contribute to a carbon neutral future” (European Commission 2018, pp. 5–6). The dominant narrative is characterized as ‘technology-driven’ and ‘biomass-based’ (Bugge et al. 2016). In this vision, a key role is assigned to centralized, large-scale high-technology industry, for which the BPM in Äänekoski is a showcase project. Within this contested field, visions of a socio-ecological transformation or of an agro-ecological bioeconomy advocated by Non-Governmental Organizations (NGO) and by critical academia merely form a theoretical and rhetorical counter-narrative (Hausknost et al. 2017).

From a critical standpoint, a transition toward bioeconomy is regarded not mainly as a question of technical feasibility but also as one of political and societal negotiation (Lühmann 2020; Vivien et al. 2019). Critical engagement with the concept and policies dates back to the 1970s, when, on the basis of his flow-fund-model, Nicholas Georgescu-Roegen criticized the everlasting economic expansion that capitalist and bio-based economic activities rely on (Giampietro and Funtowicz 2020, p. 64). The current vision and implementation of bioeconomy stand in opposition to the origins of the term ‘bioeconomics’ as coined by Georgescu-Roegen (Georgescu-Roegen 1971). They often engage with environmental concerns only at the rhetorical level (Kleinschmit et al. 2017) and do not adhere to “strong sustainability” goals as articulated in the context of the socio-ecological transformation (Gawel et al. 2019; see also: Ramcilovic-Suominen and Pülzl 2018). Current lifestyles, patterns of production and consumption, and the accompanying levels of resource usage are “never fundamentally questioned” in EU bioeconomy strategies (Lühmann 2020, p. 8). Based on Birch et al. (2010), Hausknost et al. (2017) frame the dominant vision as “politically salient and elite-driven”, leading to a discourse premised on the idea of a “neoliberalization of nature” that views the resources the bioeconomy draws on as ‘sustainable capital’ (p. 6, 16). Hence, the dominant vision is criticized for its technological solutionism in the sense of “economics of technological promises” (Giampietro 2019, p. 143) and for aiming “less at decarbonizing society and more at substituting renewable biomass for fossil carbon” (Levidow et al. 2019, p. 14). Modern bioeconomy in the EU is fundamentally questioned as incompatible with the actual “available biophysical options” and criticized for meeting neither present nor future requirements of sustainable resource use (Hausknost et al. 2017, p. 16). Many critical analyses agree that, currently, the dominant bioeconomic visions in the EU follow a Green Growth narrative (Grunwald 2020; Levidow et al. 2019). A thorough look at funding distribution as well as quantity of projects in the field makes the hegemony of the Green Growth narrative evident (Lühmann 2020).

Following these research strands, studies of tendencies and dynamics in this emerging and contested field might prove fruitful for further critical analyses.

### The case of the Finnish forest-based bioeconomy and Äänekoski BPM

The BPM in Äänekoski is a showcase project of the centralized, large-scale high-technology industry and Green Growth vision of the bioeconomy. This case encapsulates the contradictions within the European bioeconomy: the political strengthening and the growth of the bioeconomy create and foster tensions related to the governance, use, and function of natural resources in Finland, mainly forests (BIOS 2017a; Kröger and Raitio 2017; Kellokumpu 2021). Within the Finnish forest sector, almost all processes concerning sustainability and modernization are framed as contributing to a ‘forest bioeconomy’ (Ministry of Agriculture and Forestry Finland 2019). With regards to both modern forest-based bioeconomy and ambitious climate policy, Finland plays a pioneering role within the EU (Toivanen 2021). Finland was one of the first European countries to adopt a bioeconomy strategy in 2014 (“Sustainable Growth from Bioeconomy”; Ministry of Economic Affairs and Employment Finland 2014). The forest-based bioeconomy in Finland both creates hopes “in the quest for a more sustainable future” and offers “possibilities for growth and prosperity especially due to the vast forest resources available” (Peltomaa 2017, p. 57). Announcing the goal of a “carbon–neutral welfare state” until 2035, Finland is going far beyond the common European goals of the Green Deal (Ministry of the Environment Finland 2020). Bio-based modes of production as well as bio-based products and consumption practices are presented as Finland’s contribution to reducing CO_2_ and combating climate change (Lindstad et al. 2015). The “National Forest Strategy of Finland 2025” (Ministry of Agriculture and Forestry Finland 2019) complements the bioeconomy strategy through a significant rhetorical shift toward sustainability, climate change, and biodiversity. The recently updated “Finnish Bioeconomy Strategy—Sustainably Towards Higher Value Added” (Finnish Government 2022) continues on the same path.

The Finnish strategy and policies also match the dominant growth-oriented vision of bioeconomy in the EU (Albrecht 2019; Bosman and Rotmans 2016; Toivanen 2021). The current modernization of the forest sector in Finland leads to critical environmental, social, and political effects: Kröger and Raitio (2017, p. 6) show that beneath the surface of a “more of everything” framing, Finnish forest policy pairs up with the global bioeconomy discourse, resulting in, first and foremost, increased timber production and the promotion of a productivist forest industry. The question of “whether the forest-based bioeconomy is merely a rhetorical reframing of the traditional order” has been brought up and lack of public participation in the top–down approach has been criticized (Peltomaa 2017, p. 58; see also: Bosman and Rotmans 2016; Mustalahti 2018).

The Finland-based, long-established, globally operating forest and pulp/paper company Metsä Group is one of the three big players in the Finnish forest and paper industries. They aim to achieve high sustainability targets oriented toward climate neutrality, and the SDG and the company claims to redesign its entire value chain accordingly (Metsä Fibre 2022b). The company has been operating in Äänekoski since the 1960s. In 2015, they announced the biggest investment in the history of the Finnish forest industry: 1.2 billion EUR to construct a new factory on the long-standing industrial site in Äänekoski (Metsä Group Press Release 2017). The municipality of Äänekoski is the so-called typical ‘Finnish forest town’: since the end of the nineteenth century, when the first sawmill was built on the shore of the local lake, the development of the municipality and the region have been closely linked to the development of the forest and paper industries (Albrecht 2019). In total, the municipality has just under 20,000 inhabitants and is located in a relatively rural area, with the forest and paper industries among the main employers.

Advertisement for the BPM in Äänekoski highlights that by producing energy from side streams, it will increase the renewable energy share of Finland by more than 2%. It is also expected to have a significant effect on Finnish export numbers and overall gross domestic product (GDP) (Metsä Group 2022a). The BPM directly employs 150 people. Its innovative character is advertised by Metsä Group as “creating a diverse ecosystem of bioeconomy companies” (Metsä Group 2022a). It is pivotal as one of the most prominent bioeconomy megaprojects in Europe (European Circular Economy Stakeholder Platform 2022; European Investment Bank 2015), serving as a global blueprint for highly efficient, automated, and CO_2_-neutral BPMs/biorefineries (Albrecht 2019; Metsä Group Press Release 2017).

## Extractivist tendencies and patterns as a heuristic

For a more in-depth analysis of the Finnish forest-based bioeconomy and Äänekoski BPM, this paper relies on the heuristic of extractivist tendencies, dynamics, and patterns (McKay 2017; Tittor 2021). This section introduces four dimensions of the analysis that comprise extractivist patterns and tendencies based on key conceptualizations of the term that have been developed in the literature, in particular by scholars from Latin America (e.g., Acosta 2013; Gudynas 2019; Ramírez and Schmalz 2019; Svampa 2012). The concept of extractivism is linked to an “economic model or accumulation strategy that relies on the extraction of raw materials”, mainly non-renewable resources such as oil or coal from the Global South that are then exported for production and consumption in the Global North (Tittor 2021). However, the directions of trade are not limited to the well-researched South to North direction, as Asian countries are changing the dynamics and directions of global trade (Gudynas 2019; Rüland and Rodríguez 2020). Recently, new approaches have labeled extractivism a globally applicable concept and linked it to capitalism, economic growth, and unsustainability: these approaches include studies of mining or forestry activities in Canada and Finland (Kröger 2020; Stammler and Wilson 2016; Willow 2019), and emphasize perspectives on the overall extractivist logic of abstract energy (Dunlap and Jakobsen 2020) and on the industrial, large-scale extraction of renewable resources in industrial agriculture (McKay 2017; Tittor 2021) or forestry (Ehrnström-Fuentes and Kröger 2018). While so far not in the focus of these studies, the pulp industry in Finland is a named example (Chagnon et al. 2022). From an anthropological-historical perspective, extractivism is studied as a socially dominant and destructive mindset “in which long-term environmental consequences are ignored” (Willow 2019, p. 2).

Within research on extractivism, forests mainly come into the picture from the perspective of deforestation as a consequence of livestock breeding, mining, or industrial agriculture. Hence, forests are dealt with as a victim of the extractivist (bio-based) practices and structures of other sectors (Follador et al. 2019; Tittor 2021). More recently, industrial forestry and forest-based bioeconomy have themselves also been framed as an extractivist activity (Hanacek et al. 2022; Boyd et al. 2001). Case studies investigate pulp investments and plants in Uruguay (Ehrnström-Fuentes 2019; Ehrnström-Fuentes and Kröger 2018), and the intensive forest industry in Chile that threatens local livelihoods and water resources (Landherr et al. 2019), or logging in Canada and the US (Willow 2019). To the best of my knowledge, there is not yet an empirical case study applying this approach to the Finnish forest sector and Äänekoski BPM.

Research on extractivist tendencies and patterns in a global bioeconomy is still rather scattered, even though some recent studies on extractive practices in the agricultural and forest-based bioeconomy in the Global South point in this direction (Anlauf 2022; Backhouse et al. 2019; Backhouse et al. 2021; Tittor 2021): despite references to sustainability and renewability, the cases of bioeconomy studied rely on heavy extraction of natural resources and might even increase it (cf. Backhouse et al. (2019) regarding the case of sugar cane). The critical research by Tittor, Backhouse, and others suggests that the conceptual foundation extractivism is built upon might stay intact within modern bioeconomy: above all, nature is regarded as a commodity that is made to work “harder, faster and better” (Boyd et al. 2001, p. 565).

Following these research strands, this paper understands extractivism as “any economic sector (i.e., farming, fishing, and forestry) engaged in the large-scale extraction of unprocessed natural resources” (Ehrnström-Fuentes and Kröger 2018, p. 197; see also: Gudynas 2019). As a consequence of extractivist exploitation, renewable resources, such as forests or plants, might become “non-renewable” (Acosta 2013, p. 62). In line with this understanding, and following Boyer (2019, p. 178), this paper scrutinizes bioeconomy less as an alternative to extractivism and instead as a potential *alternative form* of extractivism. The following four dimensions, derived from the existing literature, guide an analysis of economic, political, social, and environmental aspects of extractivist patterns and tendencies in Finland: Dimension A: export orientation and low degree of processing, Dimension B: increasing scale, scope, and speed of extraction, Dimension C: (contradictory) socio-economic and environmental impacts, and Dimension D: extractive relations to nature. The introduction of each dimension in the following sections  provides the analytical tools for the case study in the section “Analysis of extractivist tendencies in the Finnish forest-based bioeconomy”.

### Dimension A: export orientation and low degree of processing

Extractivist tendencies in a sector or in an entire economy are characterized by a high orientation toward global markets and a large share of exported goods. Investments, innovation, and the development of standards are directed toward the global markets and their needs. This tendency includes long-distance transportation of resources that are often extracted at peripheral locations and processed in central economies (Willow 2019; Hafner et al. 2016; Dietz and Engels 2017). According to Gudynas (2019), extractivism can be analyzed as a (g)local phenomenon affected by interlinkages between globally operating corporations and local effects on society, nature, and people. In addition, degrees of processing often remain rather basic in the country or region of extraction (Gudynas 2019; McKay 2017).

### Dimension B: increasing scale, scope, and speed of extraction

Extractivist resource removal is characterized by very high volumes and high intensity as well as an increasing speed of extraction. Due to rising global resource demands and technological innovation, as well as industrial centralization tendencies, the scale, scope, and speed of extractive activities tend to increase and are leading to the intensification of external side effects (Gudynas 2019; Willow 2019; Dietz and Engels 2017; McKay 2017).

### Dimension C: (contradictory) socio-economic and environmental impacts

Socio-economic analyses of extractivist cases encompass both positive and negative patterns and tendencies: increasing social injustice and inequality are identified as one consequence of extractivism in both the Global North (Willow 2019) and the Global South (e.g., Acosta 2013; Dietz and Engels 2017; Landherr et al. 2019). At the same time, increasing job opportunities and a projected rise in living standards progressively legitimize recent neo-extractivist activities in South America (Hafner et al. 2016; Tittor 2021). They are also characterized by state support and involvement, as well as the promise of positive prospects for ‘underdeveloped’ areas (Dietz and Engels 2017; Ehrnström-Fuentes and Kröger 2018); the same holds true for bioeconomy, which is promoted as an engine for rural development both in Europe (European Commission 2018a; Wesseler and von Braun 2017) and Finland in particular (Ministry of Economic Affairs and Employment Finland 2014). Besides, extractivist tendencies are often linked to changes in norms and reforms of institutions (Dietz and Engels 2017). Analyses of environmental aspects identify soil degradation, biodiversity loss, or pollution of water, air, or soil as negative side effects (Landherr et al. 2019; McKay 2017).

### Dimension D: extractive relations to nature

Extractivism is characterized by a perception of nature as a freely available “input (e.g., a resource like oil, soil, or trees) for the production of a commodity (e.g., gas, food, or timber)” (Almeida 2020). Gudynas (2019) compares this perception of nature to ‘a faith’ that does not allow any general questioning of the sense or necessity of resource removal and use. Whereas the ‘how’ form, scale, scope, and speed of resource extraction are core concepts of research on extractivism, the collective cognitive (in)capacity to imagine nature as something that might be 'valuable' without being explicitly ‘of use’ to society or economy, should be key to the critical study of extractivism. Often, comprehensive critique is prevented, and negative environmental external effects are consequently underestimated. From her case studies of industrial forestry in Canada and the US, Willow (2019) goes as far as to describe a form of ‘cultural clear-cutting’ as an inherited relation to nature resulting in current extractivist attitudes and activities. In the bioeconomy case, valorization and commodification of nature are ongoing as more and diverse parts of ‘nature’ become ‘resources’ by being incorporated into commercial activities (Birch et al. 2010).

## Analysis of extractivist tendencies in the Finnish forest-based bioeconomy

Based on this heuristic framework, this section analyses to what extent the forest-based bioeconomy in the case of the Finnish forest industry and the BPM in Äänekoski exhibit extractivist characteristics that undermine its sustainability prospects. The analysis follows the four dimensions introduced in the section “Extractivist tendencies and patterns as a heuristic”. It is based on primary and secondary data on Finnish forests, the forest sector, and the bioeconomy as well as non-academic sources, such as reports, brochures, and websites. The author’s own qualitative data complement the analysis. In particular, dimensions C and D include subjective interviewee perceptions from Central Finland and Äänekoski (Tables 1 and 2).

**Table 1:** Interview﻿ guideline

For the analysis, mainly of Sect. “C: (Contradictory) socio-economic and environmental impacts” and section “D: Extractive relations to nature”. A selection of interviews from two field trips in 2019 (F1) and 2020 (F2) are taken into consideration. The full sampling of 16 interviews from Äänekoski and Central Finland (5 from F1; 11 from F2) was scanned regarding the aspects discussed in this paper (mainly extractivism and ‘relations to nature’) and 9 interviews incorporating diverse views and stakeholders from the sampling (male/female, different professional backgrounds, different kind of involvement in city affairs and forest industry) were selected to be taken into consideration for the analysis at hand. The explorative analysis of the interview transcriptions was based on a qualitative content analysis using the method of habitus hermeneutics (Lange-Vester and Teiwes-Kügler 2013) for group discussions among my colleagues and selective in-depth case reports for the interviews. The incorporation of interview quotes does not represent my full sampling nor aims at anything like representation, rather it is meant to provide in-depth perceptions and locally rooted aspects for the discussion	
Main question for the block	Additional questions
Could you tell me a little bit about yourself and your personal background?	What do you do for a living? // What exactly is your position and your job about? Within your daily life, can you describe to me a typical working day/week? So that I can imagine what your job is about and what you do in a normal day, like how do you go to work, how long do you work? Can you tell me something about important stages and developments in your life? Things like moving, your study or education, starting a family? What do you normally do in your free time, like on holidays, weekends or also in the afternoons? Like, how do you spend the time you are not at work? What do you do? Could you tell me a little bit (more) about your family and your childhood: Where and how did you grow up? How can I imagine your childhood?
If I come to Äänekoski for the first time, what do I have to know about this place? What is there to know about this place?	To you personally, what is the best thing, and what is the worst thing about Äänekoski? Do you like the landscape around Äänekoski? What do you not like about it? What are the biggest changes that have taken place in the town/region in the past 10 years? What role does the forest and paper sector play in Äänekoski? Did that role change over time? Have you heard about the plans of Äänekoski to become a carbon–neutral city until 2025, and what do you personally think about it?
When did you first hear about Metsa’s company’s plans to build this new mill? What did you think about it when you first heard of it and what do you think about it now?	Did the recession of the forest sector in the early 2000 had an impact on you—or on your family/friends, or on the town? Were there any conflicts or controversies about the mill in the past or also nowadays? For instance, with between the mill/Metsa Group and local politics and interest groups? What is your personal relation to the mill or to Metsa Group? Did the forest sector play a special role now during Corona crises or do you also remember something similar for earlier times of economic recession? Just out of curiosity: Have you been to Pro Nemus visitor center already or to the new mill?
I know that there are a lot of discussions in the public and in the media in Finland about forests, their management and the forest industry. What are your thoughts on that?	How would you describe the influence of the forest owners/forest industry on the forest? Like really practical: What do they do there, how do they treat the forest? What do you personally use the forest for? What do you do in the forest? And what role does NATURE in general play for you and for your life? Like not just the forest … When you think about the landscape around Äänekoski, like the forests, but also the fields and lakes—do you think that it should stay as it is right now? And when you think about your childhood, how did the landscape and nature look like? What was different from now? There are a lot of debates about the role of the forest and the forest sector for ecological sustainability and carbon neutrality—what is your opinion on it? Have you heard of the concept of bioeconomy/circular economy? Do you think that the forest sector and the forest standings have to grow also in the past in order to survive and stay prosperous? How should it be in your opinion? What do you hope for? And 10 years from now into the future, what do you think will have changed in the region and Äänekoski?
Do you have anything that you would still like to tell me? Maybe something I forgot to ask or that just came to your mind right now?

**Table 2:** Interviews﻿ incorporated into the analysis

Abbreviation	Gender, position
F1_IP16	Male, employee regional council of Finland, Jyväskylä
F2_IP1	Male, employee local public cultural institution, Äänekoski
F2_IP2	Female, employee Metsä Group, Äänekoski
F2_IP3	Male, local entrepreneur, Äänekoski
F2_IP5	Male, member of an environmental group, Äänekoski
F2_IP6/7	Female, local politician, Äänekoski
F2_IP11	Female, employee Metsä Group, Äänekoski
F1_IP20	Female, employee Metsä Group, Äänekoski

“F1”/“F2” specifies the field trip the interview material was taken from; F1 = 2019, F2 = 2020; “IP” specifies the Interview Partner’s number to identify the interview

### A: export orientation and low degree of processing

The forest-based bioeconomy in Finland, and the pulp and paper industry as part of it, is highly oriented toward global markets, global standards, and global consumption habits, and it depends on their development. The main forest industry export product is pulp and paper, and since the paper production crisis in the early 2000s, the forest industries’ main profit source has been unrefined cellulose. The Ministry of Agriculture and Forestry of Finland (2022) states that “relative to its size, Finland has the most forest-dependent and forest sector-reliant national economy in the world”. The EU market is the most important export destination for the Finnish forest industry (accounting for more than 50%), followed by Asia (almost 20%, China: 4.2%) (Finnish Forest Industries 2017, 2020a; The Observatory of Economic Complexity (OEC) 2022). 16% of the Finnish economy and almost 30% of Finnish exports are attributed to bioeconomy, of which the forestry sector amounts to the largest and the most economically important share (Luke 2021a). The majority of products in the forest industry are produced for export (Finnish Forest Industries 2016). Pulp and paper products (such as single-use products like hygienic paper products or cartons for online trade) together comprise the main export goods of the Finnish forest industry (Finnish Forest Industries 2021a). Most of the wood harvested domestically goes into that industry branch (Finnish Forest Industries 2021b).

The BPM in Äänekoski has an enormous impact on Finland’s economy and exports, because both are increasing by 0.5 billion € per year due to the mill (Metsä Fibre 2021, p. 14). Overall, the majority of the pulp produced at the BPM is for export, with the main destinations being Europe and Asia (total: 1.3 Mt/a; for export: 800,000 t/a) (Metsä Group 2022a). The importance of the factory and the sector is also highlighted by local stakeholders. An interview partner from the Regional Council of Finland stated that the “*export euros come from the bioeconomy, from pulp and paper*” (F1_IP16).

To sum up, regarding the forest-based bioeconomy in general, and Äänekoski BPM in particular, increasing extractive tendencies in the dimension of export orientation to global markets, as well as a rather low degree of refinement with a focus on pulp products, could be observed.

### B: increasing scale, scope, and speed of extraction

If the current plans are realized, the scale, scope, and speed of wood removal, processing, and transportation in Finland can be expected to grow even more in future, especially in the most forestry-dependent rural regions of the country. Finland is the most forested country in the EU; 71% of its land area is covered by forests, accounting for around 10% of Europe’s forests (European Commission 2018b). In recent years, “the annual increment has exceeded the annual fellings by 30%” (Ministry of Agriculture and Forestry Finland 2020), and the carbon stock in above-ground biomass has grown as well (around 1%/a) (Prins 2020). At the same time, Finland’s forest resources are not limitless, and due to bioeconomic innovations, the demand for wood is steadily increasing, raising the question of its ‘non-renewability’ (Eyvindson et al. 2018). Recent reports show that log removals hit a new record in 2021 with 76 million m^3^ consumed by the forest industry and for energy generation (Luke 2022). For the first time in 60 years, the annual increment of the growing stock decreased from 2014 to 2020 (Luke 2021b). The majority of the wood harvested and processed in Finland ends up in the pulp industry, and most of the gross value of the Finnish forest industry is coming from this industry branch (Finnish Forest Industries 2020b, 2019). The domestic demand is expected to grow (further) in the coming decades as a result of the innovation-driven bioeconomy strategy that foresees greater investments in the forest industry (Heinonen et al. 2020).

The economic significance of the forest sector is comparatively high in Central Finland (Mäntyranta 2019). It also is one of the regions in the country with high felling rates (Luke 2019). In the case of the BPM, the ‘superlative’ character of the plant (Albrecht 2019), its high efficiency, and the large amounts of material that enter it daily meet with the characteristics of dimension B. In comparison to the former mill, not only has the economic output increased, but also the demand for wood has risen significantly from 4 to 6.5 Mm^3^/a (Metsä Group 2022a). The local population is very aware of the increased cuttings, with several interview partners raising concerns about them (F2_IP3; F2_IP6/7).

Hence, regarding dimension B, extractivist tendencies can be identified as the demand for wood is rising, felling rates are increasing and investments and overall outputs are growing. All aspects point toward further speed and growth.

### C: (contradictory) socio-economic and environmental impacts

At the first glance, positive socio-economic effects of the forest-based bioeconomy in Finland prevail. Closer inspection, however, shows ambivalent or even negative effects, and multiple critical aspects. Despite local authorities and businesses engaging in communication efforts, the sector is criticized for a lack of participation and a high concentration of power. Developments regarding environmental aspects such as biodiversity are also met with concern. In general, the level of environmental protection is rather high in Finland, especially when considered from a global perspective; nevertheless, the local materialization of forest-based bioeconomy causes an ongoing intensification of environmental threats.

#### Economic and rural developments

Bioeconomy and investments in the modernization of the forest industry are part of Finland’s growth strategy (Ministry of Economic Affairs and Employment Finland 2014). A comparative study of industrial sectors and their energy performance (Velasco-Fernández et al. 2018) makes it clear that the pulp and paper sector in Finland, compared to other sectors, in fact, does not generate much employment, while being rather energy intensive at the same time. Technical investment per worker is also the highest of all the sub-sectors studied (Velasco-Fernández et al. 2018). This shows the extreme intensity of the sector (and, to some extent, already does point to the necessity of an extractivist relation to its main resource—the forest). The forest industry accounts for a fifth of the overall industrial production (Finnish Forest Industries 2019) and is the rural sector in Finland (Lindstad et al. 2015; for Central Finland, see: Keski-Suomen Liitto 2022).

In the past few decades, the municipality of Äänekoski has suffered from serious economic decline, growing unemployment, and a decreasing population (Albrecht and Kortelainen 2020). In the case of the BPM, unemployment in the municipality dropped significantly during the construction phase of the plant, while the long-term effects are less significant (Albrecht and Kortelainen 2020). Overall, the financial situation of the municipality has improved since the announcement of the new plant, and investments have been made in the local infrastructure (Albrecht 2019). Due to the investments and the new plant, a spirit of optimism has emerged since 2015, at least among business, political and official stakeholders. The development company for Äänekoski founded the ‘Plänet B’ project that aims to set up a local network for bio-based businesses, cooperating with various educational institutions and fostering the ‘green’ image of the city (Plänet B 2022). Confirming the findings of Albrecht and colleagues, an interview partner from the Regional Council of Finland also highlights the bioeconomy’s importance for the regional economy: it is expected to “*create economic well-being or jobs to the region or at least maintain the existing one*.” (F1_IP16). Local stakeholders involved in regional and economic developments see investment in the bioeconomy as a necessity, partly due to a lack of alternatives (F2_IP2; F1_IP16). Researchers, however, also question its sustainability from a social and environmental point of view (Albrecht and Kortelainen 2020; Albrecht 2019).

#### Participation and power

Empirical studies on the Finnish bioeconomy have shown a lack of participation as well as a concentration of power in the sector (Korhonen et al. 2018; Kröger and Raitio 2017; Mustalahti 2018; Peltomaa 2018).

In the past, the city of Äänekoski and the local mill were described as almost interchangeable, leading to non-transparent power relations and decision-making. For example, the factory manager was also the head of the local fire department, or funded local sports clubs, as a representative of a local public cultural institution mentioned in the interview (F2_IP1). Still today, local authorities and stakeholders highly value the BPM, and vice versa (F2_IP2; F1_IP20/F2_IP11). Regarding the current practice, interviewees mentioned that the communication by the BPM’s officials mainly aims to inform the public about events or changes that are already underway (e.g., with the *Pro Nemus* Visitor Centre at the premises of the BPM that invites the local population in on special occasions) (F1_IP20/F2_IP11; F2_IP3).

#### Environmental impacts

Besides the socio-economic aspects, it is necessary to consider the negative impacts of forest-based bioeconomy on biodiversity (Apostolopoulou et al. 2014; Otero et al. 2020) and a decreased capacity of forests to absorb CO^2^ (BIOS 2017b; Kellokumpu 2021). As shown above, the demand for wood processing is increasing (Luke 2019). The debate about carbon sinks and storage in Finnish forests is almost as old as the political project of the forest-based bioeconomy itself, and equally controversial (Toivanen 2021). A 2017 public statement by more than 60 researchers (BIOS 2017a), many scientific publications (BIOS 2017b; FCCP 2019; among others: Sievänen et al. 2014; Soimakallio et al. 2016), and some media reports (Frilander 2019; Teivainen 2019; Raitio 2019) criticizes the official calculations and the claims of sustainability by advocates of the forest-based bioeconomy. By highlighting the dependence on the time-frames, the calculation models used, and the annual yields, these reports point out that forests might even become a source of carbon in the future. Within critical academia, it is agreed upon that “the maximization of forest growth does not lead to a maximization of carbon sinks, and increased felling is detrimental to mitigation goals” (Kellokumpu 2021). Regarding forest biodiversity in Finland, a study by Eyvindson et al. (2018) shows that at current harvesting levels in boreal forests, high ecological costs are already obvious, in particular, because of suffering forest species. The authors emphasize that “increasing forest harvest level to the maximum economically sustainable harvest will harm biodiversity and non timber ecosystem services” (Eyvindson et al. 2018, p. 123). Similarly, the Red List for Finland (Hyvärinen et al. 2019) clearly states the dilemma concerning the forest-based bioeconomy and biodiversity, naming forestry as one of the major reasons for decreasing biodiversity. Forest biodiversity is no longer declining as rapidly as before, but the overall declining trend has not yet been halted (Convention on Biological Diversity 2022).

In the case of the BPM, most of the processed wood is harvested in regional forests in Central Finland and surrounding regions; one argument for the investment in Äänekoski was its suitable location in the middle of the forest (Metsä Group 2022a). Studying forest biodiversity in Central Finland, Bjorklund et al. (2020) conclude that it is likely that “increasing logging pressures and shorter rotation periods” pose the greatest threat in the future. Furthermore, the BPM represents a bioeconomy vision connected to an ever-increasing demand for wood that is criticized as threatening the future of forests themselves (BIOS 2017a). With its next big bioeconomy project set to be realized by 2023 in the Finnish city of Kemi, Metsä Group is again aiming to “source the pulpwood for the mill mainly from Finland” and will conduct “further studies to maximize the share of domestic wood” (Metsä Group 2022b).

Regarding dimension C, an analysis of the three elements—economic and rural developments; participation and power; environmental impacts—shows a complex picture of socio-economic and environmental aspects. Ultimately, negative environmental impacts regarding biodiversity and the capacity of forests to absorb carbon dioxide threaten the overall sustainability of forest bioeconomy. Hence, extractivist tendencies prevail in this dimension.

### D: extractive relations to nature

Dimension D entails an explorative discussion of the extractive relations to nature that interview partners witnessed in Äänekoski. Äänekoski has been and still is (or wants to be) a so-called ‘industrial forest town’, with the processing of wood playing an important role for the town (Albrecht and Kortelainen 2020). Local stakeholders regard the new ‘industrial’ as ‘clean’: under normal conditions, there is less smell, noise, and steam than before. The (historically grown) self-perception of the town as an industrial town has persisted, leading to a city planning that aims to develop the town into a bioeconomy hub and regional business center. The local population’s overall acceptance of these plans is due to the recent improvement of living and environmental conditions for most of the local population, among others (Albrecht and Kortelainen 2020).

The forest industry is not seen as a foreign or distant actor ‘infiltrating’ local grounds. In fact, the opposite is the case: it is perceived as part of the community, or even the family; it has been in the town for decades and is appreciated for bringing improvements with it (F2_IP5; F2_IP6/7). The interviewees’ links to the industry are complex and often date back to their grandparents’ or great-grandparents’ generation. The forest industry and the commercial use of forests are rooted in local livelihoods. Nature is perceived as rather robust and is taken for granted to a certain extent. Only during the exceptional (crisis) situation caused by the COVID-19 pandemic, did some people become conscious of the surrounding nature and started cherishing it (F2_IP2; F2_IP3)?

Interview partners assessed the current situation by comparing it to an unspecific past that is perceived as worse than the present, according to the motto: ‘it is better now than it has been in the past, so, we do not complain’. This is applied to communication and transparency aspects (F2_IP2; F1_IP16, F1_IP20), as well as to the development of pollution and emissions (F2_IP2; F1_IP16, F2_IP5). When mentioned in the interviews, opposition to the BPM and accompanying developments was never absolute. It was directed toward a specific aspect or a negative effect having a direct impact: increased truck traffic (more than 240 trucks per day pass through) (F2_IP3; F2_IP6/7, F2_IP5) and clear-cuttings close to roads or residential areas (F2_IP3; F2_IP6/7). “*People are confused: they need the jobs, they need the new developments and investments, but they also see the many trucks [*that go to the mill*]. …* *It does not feel good to see all that wood going into the factory every day. It does not feel right*.” (F2_IP3). Some local interviewees, acknowledging more fundamentally problematic aspects of the current developments, characterized them as unsolvable problems (F2_IP5), a dilemma (F2_IP3), or a matter with no alternative (F2_IP6/7). Many perceptions fit with a narrative of progress (‘Plänet B project’; F2_IP2; F1_IP20): a certain level of destruction of nature seems to be accepted as ‘part of the deal’.

The analysis of dimension D shows a possible basis for acceptance of extractivist practices as they are connected to local economic progress and tradition. Extractivist tendencies might continue to depend as they are not fundamentally challenged or opposed by local actors.

## Discussion

The analysis of the case of the Finnish forest-based bioeconomy, with the BPM in Äänekoski being one of its most modern and future-oriented projects, suggests that extractivism is enabled by multiple patterns and tendencies. A sustainable transition is hindered rather than fostered. Regarding dimension A, it was shown that the export orientation of the forest industry and its importance to the national economy are high. As pulp is one of the most important export products, the degree of processing remains low. Dimension B showed that the degree of fellings is increasing due to growing demand and efficiency gains as part of the investments in bioeconomy. Especially, in rural areas such as Central Finland, the effects of increased logging might increase further due to local (bio-)economy path dependencies. Regarding dimension C, the overall (local) social and economic impacts of recent developments appear rather positive. However, the general expectations in this regard are not set very high. If they exist, they are mainly about maintaining the status quo. Regarding environmental aspects, a negative picture prevails, highlighting the ongoing reconstruction of forests for economic purposes that endanger biodiversity and diminish the carbon sink capacity of the intensively managed and logged forests. Dimension D explored the expansion of bioeconomy activities building on the social tradition of forest usage as well as the deep rootedness of the industrial use of nature in Äänekoski. Recent developments are not perceived as ‘new’, and they are regarded as a faster, more efficient, cleaner, and high-tech (= ‘better’) version of what people are used to and have been involved in for decades. The BPM does not question the long-established and structurally rooted relations to nature; it enforces and modernizes them toward extractivist tendencies. Overall, the analysis has shown that the forest-based bioeconomy in Finland and its showcase project, the BPM in Äänekoski, endanger the bioeconomic principles of circularity and renewability.

In Finland, business and political officials mainly follow the dominant understanding of bioeconomy and frame it as a win–win situation. Aspects of environmental risks are relatively de-emphasized by officials. The debates are limited to the *form* of extraction (clear-cutting versus continuous coverage) and the use of the end-products (value-added, circularity, sustainable utilization), while critical academia, environmental NGOs, and activists advocate for a more substantial critique. However, their positions are often marginalized. The actual environmental effects are substantially less destructive in the Finnish case than in reported cases in the Global South, as they are socially moderated and relatively contained by comparatively high legal standards—but they are unquestionably there. Regarding old-growth forests and biodiversity in particular, the most practiced clear-cutting management form, combined with the shortened growth period and the expected increase in wood demand, poses a serious threat to the environmental status of the (Finnish) forests as well as to Finland’s biodiversity targets and social livelihoods depending on intact local forest grounds. In the Global South, (post-)colonial societal structures, unequal and non-transparent power relations, and even acts of systematic violence characterize extractivisms. In the Finnish case, a long-established, hegemonic political–business coalition does marginalize alternative public opinions despite the comparatively well-functioning public participation and seemingly transparent decision-making (Korhonen et al. 2018). There is recurring evidence of those problems, specifically in the case of continuous dismissal of the indigenous (Sami) views on the matter as well as ignored resistance against extractive activities and environmental conflicts in Artic Finland (Komu 2019; Lassila 2018, 2021; Hanacek et al. 2022). The quantitatively differentiated level does not conceal that critique against extractivist practices and structures is degraded or silenced in both contexts. The level and form of modern forest-based bioeconomy possess a new and threatening aspect. The paper could show that this is not completely covered up by high standards and good governance.

The analytical framing of extractivist tendencies and patterns is a heuristic allowing a discussion of bioeconomy cases in the EU. Regarding the assessment of the potentials and limitations of the heuristic of extractivism for further critical research on bioeconomy transitions in the EU, the analysis demonstrated the importance and necessity of differentiation between the official positions of, e.g., governments and businesses on the one hand, and the often marginalized positions of critical academia and environmental NGOs or locals on the other. The need for local case studies to investigate the complex effects of political bioeconomy strategies is clear. The analytical framing of extractivist tendencies and patterns allowed for a discussion of both latent and manifest social, political, and ecological aspects, and an inclusion of both quantitative secondary data and qualitative data. It enabled a critical account of the contradictions within the forest-based bioeconomy in Finland.

## Conclusion: unsustainable tendencies in forest-based bioeconomy

This paper discusses the sustainability aspects of the forest-based bioeconomy in Finland with the example of its showcase project, the BPM in Äänekoski, by deploying an extractivist heuristic. It argues that through this lens, the Finnish forest-based bioeconomy might not contribute to, but rather undermines, sustainability goals. The analysis combined primary and secondary economic and environmental data supported by qualitative data comprising local subjective perceptions. The case study introduced a current materialization of the dominant understanding of bioeconomy (D’Amato et al. 2017; McCormick and Kautto 2013). The discussion illustrated several entry points for extractivist patterns and tendencies. By highlighting the inconsistent aspects of current developments, the paper contributes to critical academic and public debates in Finland that do not follow the official sustainability framing of the forest-based bioeconomy (among others: Albrecht 2019; Albrecht and Kortelainen 2020; BIOS 2017a; Eyvindson et al. 2018; Peltomaa 2018; Kröger and Raitio 2017; Toivanen 2021). Once more, the unsustainable aspects and effects of an “expansion of forestry extractivism” (Hanacek et al. 2022) could be demonstrated.

The analysis of four dimensions of the extractivist heuristic—(A) export orientation and low degree of processing, (B) increasing scale, scope, and speed of extraction, (C) (contradictory) socio-economic and environmental impacts, and (D) extractive relations to nature—uncovered latent and manifest social, political, and ecological contradictions within the forest-based bioeconomy in Finland and showed its immediate effects. The analysis leads to serious questions concerning the potential for bioeconomy to constitute a positive element of a socio-ecological transformation; extractive patterns and tendencies threaten the possible contribution of bioeconomy to sustainable resource use in Finland. The long-lasting extractive economic tradition, with the forest industry being the backbone of the Finnish economy, pairs up with societal norms that frame economically sustainable and efficient forest management as ‘taking good care of nature’. The discussion of relations to nature in Äänekoski allowed for scrutiny of a complex social situation that (similar to Komu’s 2019 “refusal to resist” concept) accepts possible negative consequences of the large-scale industrial activities as ‘part of the deal’. The bioeconomy policies and discourse as well as the BPM in Äänekoski are built upon a story of industrial modernization, technological solutionism, and progress as the only way to move forward with regard to climate, sustainability, and social challenges. However, the sustainability facade of the deal might crumble, given the critical levels of national forest biodiversity, and recent development in EU environmental and climate politics. Future research building upon the case study could follow up on the updated Finnish bioeconomy strategy, further and forthcoming European bioeconomy cases, and their global interlinkages. The dimension D of this paper—‘subjective relations to nature’—in particular leaves room for further qualitative analyses of local case studies.

Besides the discussion of (a lack of) contributions of the forest-based bioeconomy toward a more sustainable economy in the future, the paper aimed to assess the potentials and limitations of an extractivist heuristic for analysis of bioeconomy transitions in the EU. This heuristic provided a critical view on current economic activities and proved useful for the analysis of the case at hand. It could be suggested that the Finnish case represents a quantitatively attenuated version of the same harmful phenomenon as present in the Global South. Extractivist patterns and tendencies are detectable in the environmental, economic, and social debates and realities. The threats of environmental degradation and political marginalization might not be visible at once, but the analysis showed that manifold ecological, economic, and social aspects need to be reconsidered for a future bioeconomy as part of a socio-ecological transformation. Hidden contradictions, as well as complex local situations and their situatedness within the long-lasting relationships to nature, hint at possible revisions of the principles and practices of the modern forest-based bioeconomy. Despite the promise of contributing to a sustainable transformation and providing an alternative to fossil extractivism, the forest-based bioeconomy in Finland is rather characterized by extractivist tendencies and might be thus itself a potential alternative form of extractivism.

## Data Availability

The author confirms that the data supporting the findings of this study are available within the article.
